# On Chorea

**Published:** 1810-12

**Authors:** 


*For the Medical and Physical Journal.*

### *On Chorea.*

THE symptoms of Chorea are so marked and evident, that any enumeration of them in this place would be superfluous. The best account of this singular disease will be found in Dr. Hamilton's observations on purgative medicines. He was the first writer who recommended trusting the cure entirely to cathartics. Sydenham indeed, who has also very accurately described this malady, administered purgatives, but he also prescribed bleeding, and paregorics. "Sanguinem ex ægri brachio ad uncias septem, plus vel minus, pro ratione ætatis, educi jubeo. Die sequente vel dimidiam partem, vel quiddam amplius, (pro ratione vel ætatis, vel etiam majoris minorisve corporis, ad subeundam catharsin aptitudine) potionis purgantis communis exhibeo." "Et vespere haustulum paregoricum propino.

"Potionem istam catharticam ad tres vices alternis diebus repetendam prescribo, et haustum paregoricum isdem noctibus. Postea sanguinem rursus extrahi curo, dein ut ad catharsin, uti prius, æger revertatur. Aque ita, alternatim sanguinem mitto, et subduco alvum, donec ægro vena ter quaterve fuerit incisa, et post singulas venæsectiones toties fuerit fuerit purgatus, quoties vires ferre posse viderentur." It does not appear, however, that this practice proved very successful, and of late years practitioners have chiefly attempted to cure the complaint, by medicines of the stimulant and tonic class. The difficulty of effecting this desirable object is unquestionably, considerable, and sometimes the patient after going through all the changes of the Materia Medica, is given up to quacks or to nature.

I had, not long since, a pleasing opportunity of witnessing the good effects of Dr. Hamilton's mode of practice. A girl, aged ten years, had been severely affected with scarlet fever when three years old, soon after which, before she recovered from the consequent debility, she was attacked with chorea; at six years she had the measles, and was again seized with chorea. I understood the complaint continued several months, and at length subsided long after the medicines had been discontinued. I saw her during a fourth attack of chorea, and never witnessed more distorted gesticulation. Placing firm reliance on Dr. Hamilton's plan, I prescribed pulv. scammon. cum calomel. gr. xii. every other morning. After the first week, her mother thought the convulsive catchings of the limbs less severe, and in a fortnight she was at times altogether free from them; but she did not completely recover till she had persevered in the medicine six weeks. At first, the bowels were somewhat constipated, but after two or three doses of the medicine, which generally operated freely, nothing indicated the necessity of persevering in its use, except the continuance of the complaint.

In another case, of a girl about the same age, who was subjected to the complaint in consequence of being frightened by lightning, I pursued the purgative plan for a month, but without the least success, although the medicine operated strongly. I then prescribed small doses of Sulphas Zinci, and in the course of a week, a favourable change in the symptoms occurred, and she gradually recovered.

When a favourable occasion presents itself, I should be inclined to try the opiate friction, recommended by Mr. Ward in this Journal.

An interesting case, in which opiate friction was successful, is recorded in the *Bibliotheque Medicale* for February, 1809. "A child, aged nine years, of great sensibility and quickness, having received a slight reprimand from her father, was seized, in January 1808, with a general trembling, which was soon followed by involuntary motions of the hands and arms. Antispasmodics were employed, but the disease increasing in violence, M. Lullier, Doctor of Medicine, was called in on the 8th day. He found the arms, legs, and head of the child in conticontinual motion; the speech and deglutition much interrupted, and respiration very laborious, from the spasmodic action of the muscles subservient to those organs.

He added large doses of camphor to the anti-spasmodic medicine already prescribed, and sweetened the ptisan with syrup of piony. These remedies produced no effect, the disease in the course of a month increased to an alarming degree, notwithstanding the use of various powerful remedies, as baths, bleeding from the foot, zinc cinchona, valerian, glysters with assafœtida and laudanum. The body became emaciated, the pulse extremely feeble, and speech and deglutition almost impracticable. In this extremity, Dr. Lullier proposed a mixture of equal parts of laudanum, and Hoffman's anodyne liquor, to be rubbed on different parts of the body, and repeated every two hours. From the second friction, the symptoms abated in violence, and about the seventh or eighth day, tranquillity was completely restored to the system. The laudanum was diminished one-third, and the interval between the frictions progressively prolonged. At the end of nine days the patient was restored to her natural state, except that she continued feeble for several weeks; but has had no relapse during the space of ten months.

